# Supplementary figures and images for: Discovery of PANoptosis-related signatures correlates with immune cell infiltration in psoriasis
Source: PLoS One. 2024 Oct 31;19(10):e0310362. doi: 10.1371/journal.pone.0310362 (PMC11527320; doi:10.1371/journal.pone.0310362)

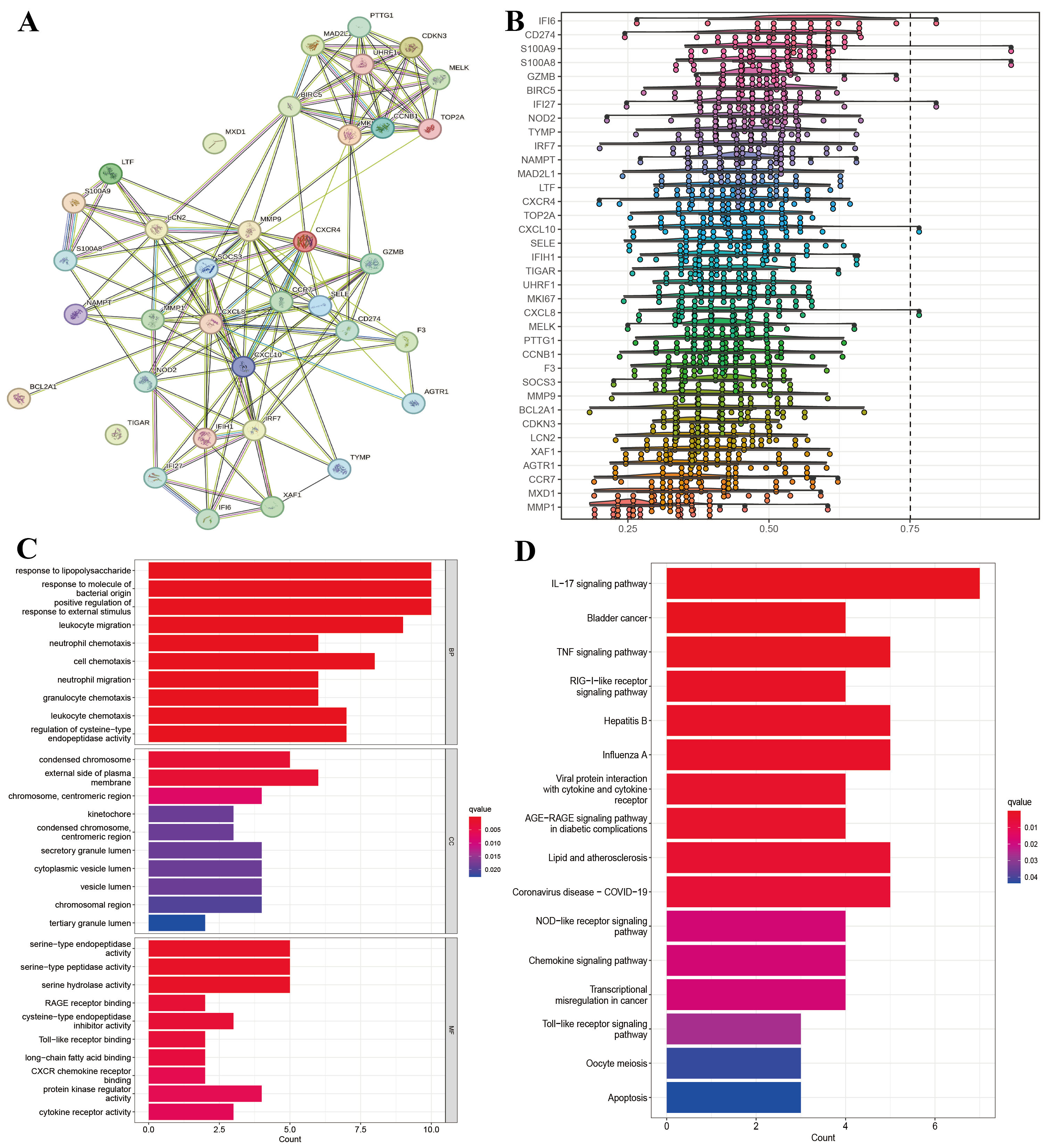

Supplement: S1 Fig — (TIF) [file pone.0310362.s001.tif]

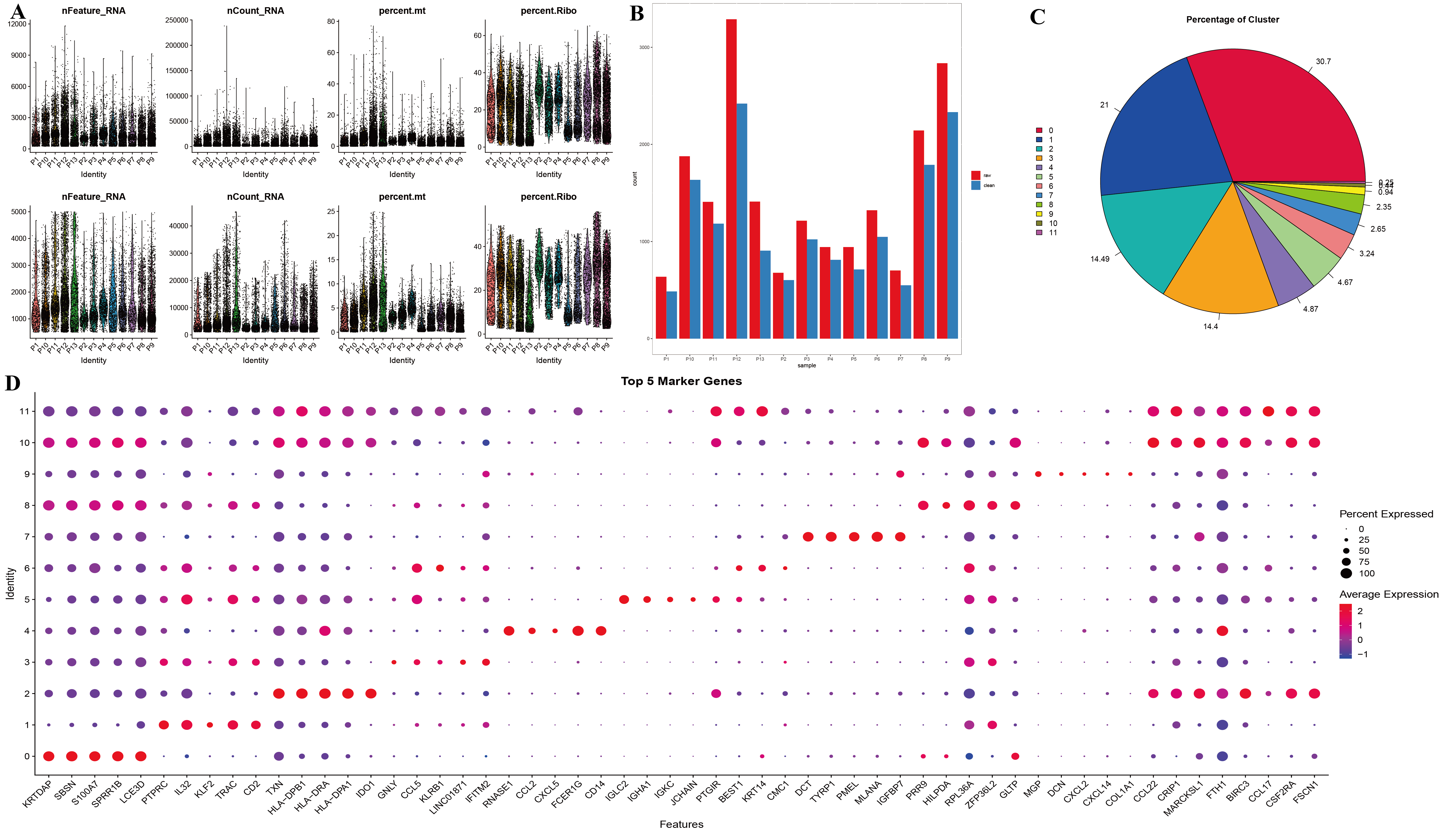

Supplement: S2 Fig — (TIF) [file pone.0310362.s002.tif]

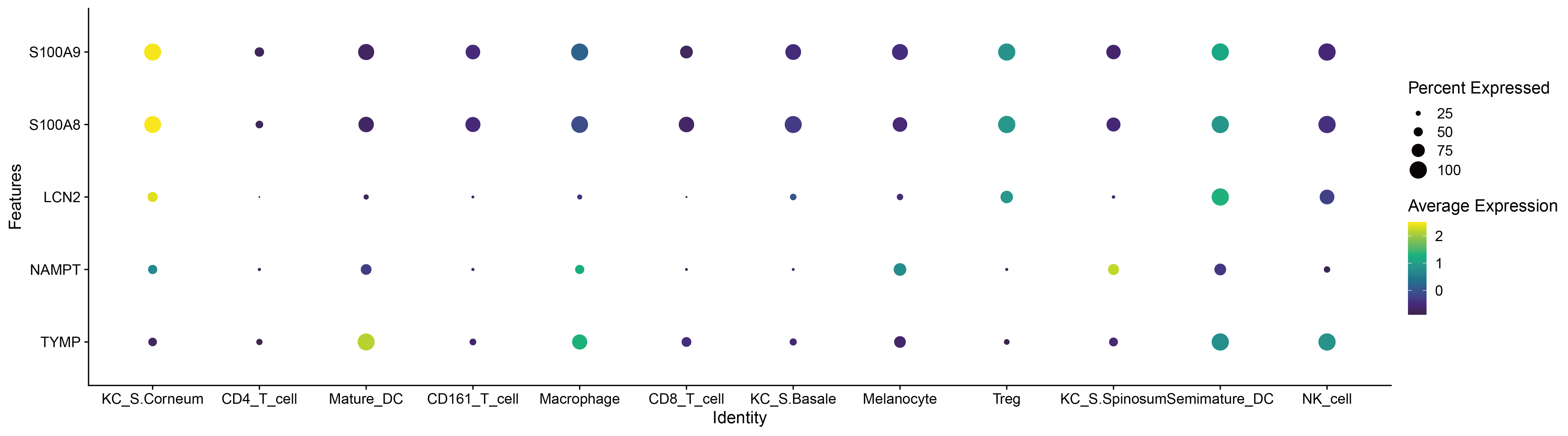

Supplement: S3 Fig — (TIF) [file pone.0310362.s003.tif]

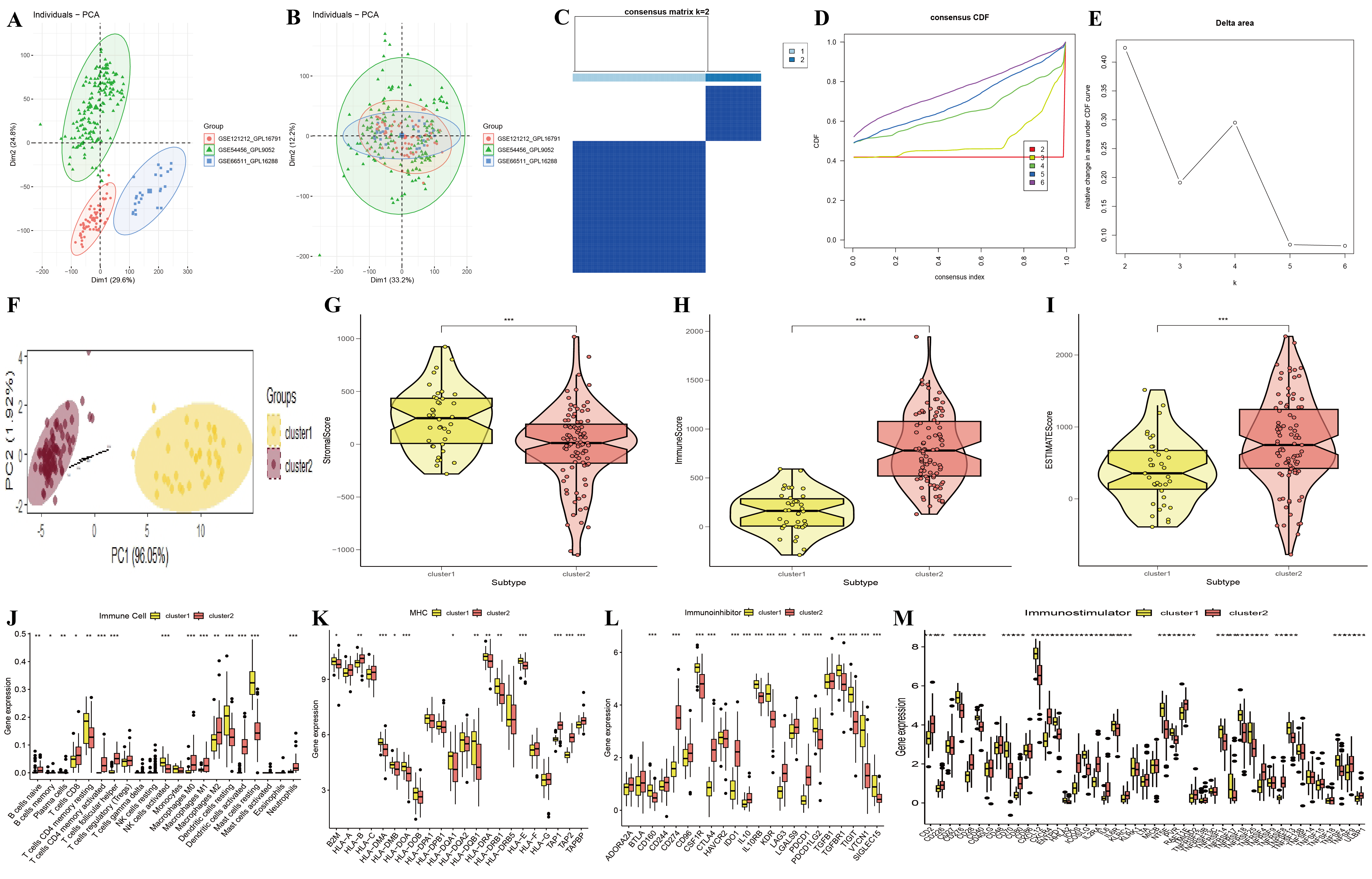

Supplement: S4 Fig — (TIF) [file pone.0310362.s004.tif]
