# Supplementary material for: Discovery of PANoptosis-related signatures correlates with immune cell infiltration in psoriasis
Source: PLoS One. 2024 Oct 31;19(10):e0310362. doi: 10.1371/journal.pone.0310362 (PMC11527320; doi:10.1371/journal.pone.0310362)
Supplement: Attachment — Submitted filename: Response to Reviewers.docx [file pone.0310362.s012.docx]

Dear editors and reviewers:

Thank you very much for your careful review of our manuscript entitled “Discovery of PANoptosis-related signatures correlates with immune cell infiltration in psoriasis”. We have carefully considered the reviewers’ professional comments and made a point to point response as follows:

**To the editor:**

1. There are major weaknesses in the methods as stated by the reviewers. The Figures' quality should be improved (pdf files are not sufficient). The Authors should clearly indicate what is new in their results as compared to those already published.

**Response：**Thank you for your valuable feedback on our manuscript. We appreciate the opportunity to address your concerns regarding the methods, figure quality, and the novelty of our results.

（1） Methodological details: In the revised manuscript, we have provided a comprehensive description of the experimental methodologies utilized in this study. We believe this clarification strengthens the rigor of our methods section. We encourage you to refer to the revised manuscript for these details.
（2） Figure quality: We acknowledge your concern regarding the quality of the figures. We are committed to addressing this and will re-upload high-quality images to ensure they meet the journal’s standards.
（3） Novel contributions: Regarding the novelty of our findings, we would like to emphasize that our research integrated multi-omics data from transcriptomics, proteomics, and single-cell transcriptomics. We employed a variety of bioinformatics approaches, including differential gene expression analysis, Weighted Gene Co-Expression Network Analysis (WGCNA), and machine learning techniques to identify biomarkers associated with PANoptosis in psoriasis. Furthermore, our study presented a novel molecular classification of psoriasis patient cohorts based on PANoptosis signature (PANoptosis-sig). This classification had been validated in our testing cohort, demonstrating reliability and stability. Significant differences in immune landscape among the distinct molecular subtypes were represented, aiding in personalized treatment strategies for psoriasis patients.

We believe these contributions represent a significant advancement in understanding the molecular underpinnings of psoriasis and can have meaningful implications for clinical practice. Thank you once again for your insightful comments, and we look forward to your further evaluation of our revised manuscript.

2. The aim of this study was to re-analyze psoriasis RNAseq data with newly developped bioinformatics tools in order to test a possible involvement of PANoptosis-related mechanisms in the disease pathogenesis. Such a study could be of interest for understanding the disease and proposition of new therapeutic targets. However, the quality of figures are not sufficient to evaluate the accuracy of the reported data. The methods were not sufficently described. I did not understand the differences between the submitted data (Fig 1) and the already published ones starting from the same GSE13355 dataset (Clin Cosmet Invest Dermatol, 2024 17:1281-95). Differential expression was considered as significant when |log2FC|>1.5, as stated in the methods section. This is apparently not the case on Fig 1A (volcano plot). Please clarify.

**Response：**Thank you for your valuable feedback on our study, we appreciate your attention to details and would like to address your concerns as follows.

Firstly, in our differential expression analysis, we employed the R package“limma”, which utilized a generalized linear model technique to detect differentially expressed genes (DEGs). We selected DEGs based on the criteria of |log2 fold change| > 1.5 and *adjusted p*-value < 0.05. We sincerely apologized for the oversight that resulted in discrepancies in the visualization of the volcano plot (Fig 1A). We have corrected the plot and uploaded the revised version.

Secondly, regarding the differences between our results and those reported in Clin Cosmet Invest Dermatol, 2024 17:1281-95, the key distinction lied in the thresholds we adopted for selecting DEGs. The study you referenced used a significance threshold of p < 0.05 and |logFC| > 0.1. Consequently, the DEGs identified from the GSE13355 dataset differed between the two analyses due to these differing criteria.

Lastly, we have enhanced the methods section in the revised manuscript to ensure a clear and comprehensive description of our analysis. We believe these improvements will adequately support our findings.

3. 33 genes compose the panoptosis signature (GeneCards database). Did the Authors considered these genes? If not (IFI6, CD274, S100A8/9, 62MB were apparently not), please explain.

**Response：**Thank you for your insightful question regarding the PANoptosis signatures and the specific genes you mentioned. In our study, we have taken into account the feedback from the editor and reviewers to improve our research methods significantly. After integrating multi-omics data from transcriptomics, proteomics, and single-cell transcriptomics, we identified new PANoptosis-related signatures(PANoptosis-sig) comprising the genes TYMP, S100A8, S100A9, NAMPT and LCN2. We validated the differential expression of PANoptosis-sig between psoriasis and healthy controls at transcriptional and protein levels. Additionally, we conducted an analysis of the relationship between PANoptosis-sig and immune cells, which adds further context to their roles in disease pathogenesis. For a detailed overview of our findings and their implications, please refer to the results and discussion sections of the manuscript.

4. The abstract is not informative about the results, it sounds like a brief description of the methods used. What do your analysis add to the understanding of the disease?

**Response：**Thank you for your professional suggestions. We acknowledge that the original abstract may have focused excessively on the methods employed rather than adequately conveying the study's findings. In response to your comments, we have revised the abstract to provide a comprehensive overview of the results obtained from our analysis. This updated abstract highlights the key findings and their implications for enhancing our understanding of the disease, specifically in relation to the mechanisms of PANoptosis in psoriasis. We believe that these revisions offer a clearer picture of our contributions to the field and better reflect the significance of our findings. For detailed insights, please refer to the revised manuscript. Thank you for your valuable input, which has helped us improve the clarity and impact of our work.

Our analysis contributes significantly to the understanding of psoriasis by elucidating the role of PANoptosis in the disease's pathogenesis. By identifying PANoptosis-related signatures (PANoptosis-sig) from skin tissue of psoriasis patients and healthy controls, we have established a novel framework for exploring the interplay between apoptosis, pyroptosis, and necroptosis in the context of psoriasis. Specifically, we discovered five key PANoptosis-sig(TYMP, S100A8, S100A9, NAMPT, and LCN2) that were critically involved in inflammatory processes associated with psoriasis. Our enrichment analysis revealed that PANoptosis-sig were linked to mechanisms such as leukocyte aggregation, migration, chronic inflammatory responses, and the IL-17 signaling pathway, all of which were central to psoriasis pathology.

The construction of predictive and diagnostic models using the PANoptosis-sig enabled us to assess the potential for these biomarkers in clinical settings. Additionally, the identification of distinct molecular subtypes of psoriasis through unsupervised consensus clustering provided a deeper understanding of the heterogeneity for psoriasis, suggesting that different subtypes might respond differently to treatments, particularly immunotherapies.

Overall, our findings not only revealed potential biomarkers and therapeutic targets for prevention, diagnosis and treatment but also enhanced the understanding of the molecular mechanisms underlying PANoptosis in psoriasis, paving the way for more targeted and effective interventions.

**To Reviewer 1：**

1. This study used quite small number of samples. Why only microarray data were used? Pleanty of RNAseq data (GSE66511) are available and using microarrays seems strange.

**Response：**We appreciate your insightful comments regarding our manuscript. We acknowledge the limitations associated with using only microarray data in our study. In response to this concern, we have included RNAseq data from the GSE66511, GSE54456 and GSE121212 datasets, as suggested.

It should be noted that the inclusion criteria for our study necessitated datasets containing both psoriatic and healthy control skin samples, with each group comprising no fewer than 20 samples of skin tissue. Consequently, our final dataset encompassed 276 psoriatic lesion skin samples and 247 healthy control skin samples. This comprehensive datasets now allowed for a more robust analysis, leveraging the strengths of both microarray and RNAseq technologies. We believe that this addition significantly enhances the rigor and scope of our study, addressing your valid concern regarding the choice of data type. We are grateful for the opportunity to improve our work based on such constructive feedback.

2. How the data were combined? The datasets that authors use are from different platforms and cannot be joined.

**Response：**We appreciate your query regarding the integration of datasets from different platforms in our study. To address this challenge, we employed the SVA (Surrogate Variable Analysis) package within the R programming environment for data integration. SVA is a well-established method for integrating heterogeneous datasets, such as those derived from microarray and RNAseq platforms[1]. This approach involves modeling and adjusting for batch effects and other sources of variability that arise from different experimental platforms. Specifically, SVA utilizes surrogate variables to capture and adjust for latent sources of variation that are not directly related to the biological variables of interest, thereby harmonizing the datasets prior to downstream analyses.

In our study, after preprocessing the microarray and RNAseq data separately according to their respective platform-specific protocols, we applied SVA to adjust for technical variations and ensure comparability across datasets. This methodological step allowed us to effectively combine the datasets while controlling for potential biases introduced by platform-specific differences. Kwanha Yu also used this approach to remove batch effect in their study[2]. Shi Zhang used SVA to merge microarray datasets and decrease heterogeneities in their studies[3]. Andrew E Teschendorff described the reliability that SVA could be used to eliminate confounders in large-scale microarray analysis[4]. So, We believe that employing SVA for data integration strengthens the validity of our findings by minimizing the impact of technical variability and maximizing the biological insights gained from the integrated analysis.

**References:**

[1]. Leek JT, Johnson WE, Parker HS, Jaffe AE, Storey JD. The sva package for removing batch effects and other unwanted variation in high-throughput experiments. Bioinformatics (Oxford, England). 2012;28(6):882-3.
[2]. Yu K, Lin CJ, Hatcher A, Lozzi B, Kong K, Huang-Hobbs E, et al. PIK3CA variants selectively initiate brain hyperactivity during gliomagenesis. Nature. 2020;578(7793):166-71.
[3]. Zhang S, Wu Z, Xie J, Yang Y, Wang L, Qiu H. DNA methylation exploration for ARDS: a multi-omics and multi-microarray interrelated analysis. Journal of translational medicine. 2019;17(1):345.
[4]. Teschendorff AE, Zhuang J, Widschwendter M. Independent surrogate variable analysis to deconvolve confounding factors in large-scale microarray profiling studies. Bioinformatics (Oxford, England). 2011;27(11):1496-505.
3. Statistical significance table is missing? What genes were differentialy expressed.

**Response：**Thank you for your professional suggestions. We apologized for the previous omission of not providing the results of differential gene expression analysis. The results have now been added to the supplementary table(S2 Table) in the latest version of the manuscript.

To conduct differential gene expression analysis, we employed the limma (Linear Models for Microarray Data) package, a widely used tool for identifying differential expressed genes (DEGs). Limma fit linear models to expression data from microarray experiments and RNAseq, allowing for robust statistical inference even with limited sample sizes. In our study, we applied limma to analyze gene expression differences between psoriatic lesion and healthy control samples in the training set. We adhered to standard procedures recommended by the limma package, including normalization, correction for multiple testing, and the determination of fold changes and associated statistical significance levels (adjusted *p*-values). The results of our differential expression analysis, a total of 256 DEGs were identified from psoriasis and healthy control samples, with 191 genes upregulated and 65 genes downregulated (Fig 1A, S2 Table). The S2 table provided a comprehensive view of the molecular changes associated with psoriasis pathogenesis, highlighting genes which were significantly upregulated or downregulated in psoriatic lesions compared to healthy skin.

4. The authors should use this tool https://geoexplorer.rosalind.kcl.ac.uk . it helps to combine several datasets from different platforms and helps to increase the power.

**Response：**Thank you for your insightful suggestion regarding the use of the GEOexplorer tool for combining datasets. We appreciate your recommendation and understand its potential to enhance data integration and analysis. In our study, we opted to utilize the SVA package in R for data merging and correction of unrelated variation. This choice was based on our familiarity with the package's capabilities and specific advantages in our analysis context. However, we acknowledge the benefits of exploring alternative tools like GEOexplorer, and we will consider incorporating insights from this platform into our future work. Your feedback is invaluable in guiding us toward more robust methodologies.

5. The abstract states that "These findings revealed potential biomarkers and therapeutic targets for the prevention, diagnosis, and treatment of psoriasis, enhancing our understanding of the molecular mechanisms underlying PANoptosis." What are these genes ? The authors do not present any genes that are differnetially expressed.

**Response：**Thank you for your professional suggestions. In our study, we had revised our analysis methods based on the feedback from the editor and reviewers, resulting in new PANoptosis signatures (PANoptosis-sig). This information had been included in the abstract of the revised version. We identified five key PANoptosis-sig that might serve as potential biomarkers and therapeutic targets for psoriasis. These genes were TYMP, S100A8, S100A9, NAMPT and LCN2. We validated the differential expression of PANoptosis-sig between psoriasis and healthy controls at transcriptional and protein levels. The enrichment analysis highlighted the roles of PANoptosis-sig in inflammation-related processes such as leukocyte aggregation, migration, and responses to the IL-17 signaling pathway. Overall, PANoptosis-sig represented potential targets for future therapeutic interventions and diagnostic tools in managing psoriasis, thereby enhancing our understanding of the molecular mechanisms contributing to the disease.

6. It is not understandable, how this study is different from any differential gene expression analysis? What are the new findngs and new results.

**Response：**Thank you for your professional suggestions. We would like to emphasize that our research integrated multi-omics data from transcriptomics, proteomics, and single-cell transcriptomics. We employed a variety of bioinformatics approaches, including differential gene expression analysis, Weighted Gene Co-Expression Network Analysis (WGCNA), and machine learning techniques to identify biomarkers associated with PANoptosis in psoriasis. Furthermore, our study presented a novel molecular classification of psoriasis patient cohorts based on PANoptosis signature (PANoptosis-sig). This classification had been validated in our testing cohort, demonstrating reliability and stability. Significant differences in immune landscape among the distinct molecular subtypes were represented, aiding in personalized treatment strategies for psoriasis patients.

We believe these contributions represent a significant advancement in understanding the molecular underpinnings of psoriasis and can have meaningful implications for clinical practice.

7. The quality of figures is low and they are illegible.

**Response：**Thank you for your valuable feedback regarding the quality of the figures in our manuscript. We acknowledge that the low resolution and illegibility detracted from the clarity of our findings. In response to your concerns, we will upload high-resolution versions of all figures to ensure they are clear and legible. We understand that high-quality visuals are essential for effectively communicating our data and supporting our conclusions. Thank you for your understanding and for giving us the opportunity to improve our submission.

8. Overall, the sample size is small for this type of study. Sample size is so small that it excludes efficient splitting it into training and experimental sets.

**Response：**Thank you for your professional suggestions. We acknowledge the concern raised about the initial sample size being insufficient for splitting into training and experimental sets. To address this limitation, we have expanded our dataset to include additional RNAseq data. The revised study now incorporates a total of 5 microarray datasets and 3 RNAseq datasets, comprising 276 psoriatic skin samples and 247 healthy control samples. Specifically, we had designated the GSE13355 dataset as the training set, while the remaining datasets had been utilized as validation sets. This approach enhanced the robustness of our model by ensuring a more reliable and generalized evaluation of our findings. For a detailed breakdown of the datasets and their allocation, please refer to Table 1 in the methods section of the revised manuscript. We believe this increased sample size will significantly improve the reliability and validity of our results, addressing the initial concerns regarding the dataset's adequacy for robust model training and validation.

9. The major revision is required, addition of RNAseq data or even using only RNAseq data. More description in the methods and gene expression tables have to be presented.

**Response：**We appreciate your valuable suggestions. We have incorporated RNAseq data analysis into our revised manuscript to corroborate our research findings. The inclusion of RNAseq data allowed us to validate our initial findings with a more robust approach, thereby strengthening the scientific rigor and reliability of our conclusions. Furthermore, we have provided expanded methodological details in the methods section and included gene expression tables in supplementary materials(https://www.jianguoyun.com/p/DZjVNUoQkbumDBiVlK4FIAA) to enhance the transparency and reproducibility of our study.

**To Reviewer 2：**

1. How does single-cell sequencing address the issue of technical variability, including the exclusion of false positive or false negative results?

**Response：**Thank you for your insightful question regarding the management of technical variability and the handling of false positive and false negative results in single-cell sequencing. The single-cell dataset(GSE151177) used in our study uses 10×Genomics technology. The 10×Genomics single-cell sequencing technology incorporates several methodologies to mitigate these issues.

Firstly, 10×Genomics single-cell sequencing utilizes a microfluidic-based approach to encapsulate individual cells into unique droplets along with barcoded beads. Each bead is pre-coated with unique oligonucleotides that are designed to capture cDNA from mRNA molecules in the cell. This approach minimizes cross-contamination between cells and ensures that each transcript is accurately associated with its cell of origin. Additionally, the integration of multiplexed bead-based technology enhances the reproducibility of results by reducing technical variability.

Secondly, to address false positives, the 10×Genomics platform employs several layers of quality control. First, the system includes stringent criteria for filtering out low-quality cells based on metrics such as the number of detected genes and the total RNA content. Cells with unusually high levels of detected genes or excessive mitochondrial gene expression are flagged as potential technical artifacts. Furthermore, the use of barcoding and unique molecular identifiers (UMIs) helps in distinguishing true biological signals from technical noise by enabling the accurate counting of unique transcripts, thereby reducing the impact of amplification artifacts. In our research, data quality control was performed separately for each sample before data integration. In order to eliminate partial cells and doublets, genes expressed in fewer than three cells, cells with more than 5,000 genes, and mitochondrial genes expressed in more than 25% by mitochondrial cells were filtered out.

Thirdly, to minimize false negatives, the 10×Genomics protocol employs a comprehensive approach to capture a broad range of transcripts, including those present in low abundance. The use of UMIs also aids in accurately quantifying lowly expressed genes by distinguishing between genuine biological signals and technical dropouts. Additionally, the high sensitivity of the microfluidic encapsulation method ensures that even rare cell populations are captured and analyzed, thus reducing the likelihood of missing important biological information.

Overall, the 10×Genomics single-cell sequencing platform combines advanced technical features with robust quality control measures to address the challenges associated with technical variability and to minimize the occurrence of false positive and false negative results. These strategies collectively enhance the reliability and accuracy of single-cell transcriptomic analyses.

2. What are the important components of PANoptosome in the Introduction? Caspase-1 and Caspase-8 are not mentioned?

**Response：**Thank you for your professional suggestions. We have modified the relevant content of PANoptosome in the artical, please refer to the introduction part of the manuscript for details. PANoptosome complexes function as molecular scaffolds for concurrently engaging multiple key molecules from the pyroptotic (NLRP3, ASC, Caspase-1), apoptotic(Caspase-8, Caspase-3, FADD), and necroptotic (RIPK1 and RIPK3) cell death pathways[5-9]. Genetic and biochemical evidence indicates that proteins within the PANoptosome can be broadly divided into three groups: (1) ZBP1 and NLRP3, which are likely PAMP and DAMP detectors, (2) ASC and FADD, serving as adaptors, and (3) CASP1, CASP8, RIPK1 and RIPK3, as catalytic effectors[10]. PANoptosis is a process with intermolecular interaction among pyroptosis, apoptosis, and necroptosis[11]. PANoptosome complexes exert scaffolds and enzyme-based catalytic modifications to induce PANoptosis.

**References:**

[5]. Zheng M, Kanneganti TD. The regulation of the ZBP1-NLRP3 inflammasome and its implications in pyroptosis, apoptosis, and necroptosis (PANoptosis). Immunological reviews. 2020;297(1):26-38.
[6]. Lee S, Karki R, Wang Y, Nguyen LN, Kalathur RC, Kanneganti TD. AIM2 forms a complex with pyrin and ZBP1 to drive PANoptosis and host defence. Nature. 2021;597(7876):415-9.
[7]. Briard B, Malireddi RKS, Kanneganti TD. Role of inflammasomes/pyroptosis and PANoptosis during fungal infection. PLoS pathogens. 2021;17(3):e1009358.
[8]. Malireddi RKS, Kesavardhana S, Karki R, Kancharana B, Burton AR, Kanneganti TD. RIPK1 Distinctly Regulates Yersinia-Induced Inflammatory Cell Death, PANoptosis. ImmunoHorizons. 2020;4(12):789-96.
[9]. Hornung V, Ablasser A, Charrel-Dennis M, Bauernfeind F, Horvath G, Caffrey DR, et al. AIM2 recognizes cytosolic dsDNA and forms a caspase-1-activating inflammasome with ASC. Nature. 2009;458(7237):514-8.
[10]. Samir P, Malireddi RKS, Kanneganti TD. The PANoptosome: A Deadly Protein Complex Driving Pyroptosis, Apoptosis, and Necroptosis (PANoptosis). Frontiers in cellular and infection microbiology. 2020;10:238.
[11]. Qi Z, Zhu L, Wang K, Wang N. PANoptosis: Emerging mechanisms and disease implications. Life sciences. 2023;333:122158.
3. It is suggested to add PCR and immunohistochemical experiments for verification.

**Response：**Thank you for your professional suggestions. We appreciate the importance of experimental verification in strengthening the conclusions of our study. Due to the current constraints in our laboratory resources and capabilities, we were unable to conduct these additional experimental validations within the scope of this study. Our research primarily focuses on the comprehensive analysis of publicly available bulk and single-cell RNAseq transcriptomes and proteomes data, which provided a robust foundation for our findings. However, we recognize the merit of incorporating PCR and immunohistochemical validation to further substantiate our results. We plan to address this in future work, contingent upon the availability of resources. In the interim, we have emphasized the reliability of our findings by utilizing well-established methodologies and rigorous data analysis techniques, as well as by cross-referencing our results with existing literature and public databases.

We hope that the detailed analysis and the rigorous data-driven approach employed in this study will provide a strong basis for the conclusions drawn, while we look forward to validating our findings through additional experimental methods in subsequent research. Thank you once again for your understanding and constructive suggestions.

4. How to remove batch differences between different data sets in the article?

**Response：**Thank you for your professional suggestions. In the article, batch differences between distinct datasets were addressed using the SVA (Surrogate Variable Analysis) package, a well-established method for identifying and correcting batch effects in high-dimensional data. The SVA package is utilized to estimate surrogate variables that capture the latent batch effects not accounted for by the known experimental design. These surrogate variables are then incorporated into the statistical models to adjust for unwanted variation, thereby mitigating the influence of batch effects on downstream analyses. Specifically, the procedure involves the following steps:

Model fitting: We initially fit a model to the data to capture the primary effects of interest while excluding potential batch effects. This step includes the use of design matrices to represent known covariates and experimental conditions.

Surrogate variable estimation: Using the SVA package, we then perform an analysis to estimate surrogate variables that capture the residual variation not explained by the known covariates. The SVA package employs empirical Bayes methods to identify these surrogate variables from the data.

Incorporation of surrogate variables: The estimated surrogate variables are integrated into the statistical model as additional covariates. This adjustment helps to control for batch effects and other unwanted sources of variation.

Validation: We validate the effectiveness of batch effect correction by assessing the residual variation and ensuring that the primary biological signals of interest are preserved.

By applying this approach, we effectively reduce batch-related discrepancies between datasets, leading to more accurate and reliable results in our subsequent analyses.

5. This paper analyzes the results from the genomics and is it possible to analyze the results from the proteomics to enhance the feasibility of the results.

**Response：**Thank you for your valuable suggestion. In response to your comment, we have incorporated a proteomics analysis into our revised manuscript. This addition allowed us to perform a more comprehensive investigation of the PANoptosis-sig associated with psoriasis. We have elucidated this signature in detail, examining its differential expression at both the transcriptomic and proteomic levels. Furthermore, we constructed machine learning diagnostic models based on the PANoptosis-sig. The performance of these models was found to be highly effective, further validating the reliability of our results. We believe these enhancements significantly strengthen our findings and offer a more robust understanding of the underlying mechanisms in psoriasis.

6. Please summarize the main findings of the study.

**Response：**Thank you for your insightful comments. In response to your request for a summary of the main findings of our study, we present the following:

Our study elucidated the role of PANoptosis-a process involving interactions among pyroptosis, apoptosis, and necroptosis-in psoriasis, an inflammatory skin disease characterized by recurrent relapses. We employed a novel approach to identify PANoptosis-related signatures (PANoptosis-sig) from skin tissue of psoriasis patients and healthy controls at both transcriptional and protein levels. We identified five key PANoptosis-sig biomarkers: TYMP, S100A8, S100A9, NAMPT, and LCN2. Enrichment analysis revealed that these signatures were primarily involved in leukocyte aggregation and migration, chronic inflammatory responses, and the IL-17 signaling pathway. Single-cell transcriptome analysis demonstrated that TYMP and NAMPT were ubiquitously expressed across various cell populations, whereas LCN2, S100A8, and S100A9 were markedly elevated in keratinocytes.

We constructed predictive and diagnostic models based on PANoptosis-sig biomarkers and assessed their performance. Furthermore, using unsupervised consensus clustering analysis, we classified the psoriasis cohort into two distinct molecular subtypes. Notably, cluster 1 exhibited higher stromal score and specific immune cell profiles, including elevated levels of plasma cells, tregs, and resting mast cells. In contrast, cluster 2 had higher immune and estimate scores, with increased expression of T cells, M1 macrophages, activated dendritic cells, and neutrophils. Additionally, Cluster 2 demonstrated greater sensitivity to immune checkpoint therapies.

Our findings provided new insights into potential biomarkers and therapeutic targets for psoriasis, advancing our understanding of the molecular mechanisms associated with PANoptosis and offering implications for the prevention, diagnosis, and treatment of this chronic condition.

7. At the end of the introduction is not clear which are the innovative aspects of the research.

**Response：**Thank you for your feedback. To clarify the innovative aspects of our research:

（1） Identification of PANoptosis-related signatures: Our study introduced a novel approach by identifying specific PANoptosis-related signatures (TYMP, S100A8, S100A9, NAMPT, LCN2) in psoriasis. This was an innovative effort to explore how PANoptosis-a process that integrated pyroptosis, apoptosis, and necroptosis-contributed to psoriasis pathology.
（2） Integration of multiomics data: We employed a comprehensive analysis combining transcriptional and translational data, along with single-cell transcriptome analysis. This multi-dimensional approach enhanced the understanding of PANoptosis in psoriasis and provided a detailed view of how these signatures functioned across different cell types.
（3） Development of predictive and diagnostic models: The research involved constructing and evaluating predictive and diagnostic models based on the PANoptosis-sig. This contributed to the potential development of new diagnostic tools and therapeutic strategies for psoriasis.
（4） Identification of molecular subtypes: We used unsupervised consensus clustering to classify psoriasis into two distinct molecular subtypes based on PANoptosis-sig. This classification revealed differing immune landscapes and responses to immune checkpoint therapies, offering insights into personalized treatment strategies.

These innovative aspects collectively advanced the understanding of PANoptosis in psoriasis, uncovering potential biomarkers and therapeutic targets that could significantly impact the prevention, diagnosis, and treatment of the disease.
